# A single-arm phase II clinical trial of anlotinib combined with chemotherapy for the treatment of metastatic triple-negative breast cancer

**DOI:** 10.3389/fonc.2023.1122294

**Published:** 2023-04-12

**Authors:** Jia-Yi Huang, Xiao-Feng Xie, Xue-Lian Chen, Qiu-Yi Zhang, Li-Ping Chen, Xue Bai, Xiao-Feng Lan, Lin Song, Jin-Feng Guo, Cai-Wen Du

**Affiliations:** Department of Medical Oncology, National Cancer Center/National Clinical Research Center for Cancer/Cancer Hospital & Shenzhen Hospital, Chinese Academy of Medical Sciences and Peking Union Medical College, Shenzhen, Guangdong, China

**Keywords:** anlotinib, angiogenesis, chemotherapy, triple-negative breast cancer (TNBC), tyrosine kinase inhibitor (TKI)

## Abstract

**Background:**

Anlotinib is a novel oral small-molecule tyrosine kinase inhibitor (TKI), which can inhibit angiogenesis. The purpose of this study was to evaluate the efficacy and safety of anlotinib combined with chemotherapy in patients with metastatic triple-negative breast cancer (TNBC).

**Methods:**

This phase II clinical trial included 40 patients with metastatic TNBC who had previously received anthracycline and/or taxane treatment. All patients received anlotinib combined with chemotherapy. The primary endpoint was progression-free survival (PFS). The secondary endpoints included overall survival (OS), objective response rate (ORR), clinical benefit rate (CBR), disease control rate (DCR) and safety.

**Results:**

During May 1, 2019 and April 30, 2022, there were 40 patients enrolled in this study. The median PFS and median OS were 8.8 months (95% confidence interval [CI] 6.5-11.1 months) and 19.0 months (95% CI, 12.1–25.9 months), respectively. The ORR, CBR and DCR were 40.0% (16/40), 85.0% (34/40) and 95.0% (38/40), respectively. Cox univariate and multivariate analyses demonstrated that having more than 3 metastatic sites (p = 0.001; p = 0.020) was an independent and meaningful unfavorable prognostic factor for PFS. 37.5% of patients had grade 3 to 4 treatment-related adverse events (TRAEs). The grade 3 to 4 TRAEs included neutropenia (22.5%), leukopenia (20.0%), secondary hypertension (10.0%), hand-foot syndrome (5.0%), vomiting (5.0%), proteinuria (5.0%) and thrombocytopenia (2.5%). None of the patients withdrew from the study or died due to TRAEs.

**Conclusion:**

In this single-arm study, the treatment of metastatic TNBC with anlotinib combined with chemotherapy showed certain efficacy, and its toxicity was acceptable.

## 1 Introduction

Among women, breast cancer is the cancer with the highest incidence rate worldwide at present, and it is also one of the main causes of cancer death. The 2020 global cancer statistics showed that there were about 2.26 million women were newly diagnosed with breast cancer, and 684,996 women died of breast cancer (1). In China, breast cancer is also the most common diagnosed cancer in females, with 429,105 new cases per year and 124,002 deaths (2). Despite advances in cancer treatment, 20% to 30% of early breast cancer patients will still relapse or metastasize (3). The median overall survival (OS) period of metastatic breast cancer is generally only 2 to 3 years (4).

Triple-negative breast cancer (TNBC) is defined as the absence of estrogen receptor (ER) and progesterone receptor (PR) expression and non-amplified human epidermal growth factor receptor 2 (HER2) expression; it accounts for about 12-20% of all invasive breast cancers (5–7). TNBC has a poor clinical prognosis, and has the characteristics of highly heterogeneous, strong invasion and high degree of malignancy. It is prone to recurrence and metastasis. The most important systemic treatment of TNBC is chemotherapy, however, the effective rate of chemotherapy alone is unsatisfactory.

Angiogenesis is a key factor in the processes of growth, invasion and metastasis of malignant tumors (8). Therefore, antitumor angiogenesis strategies can be used as an effective means to treat cancer (9, 10). Anti-angiogenic drugs mainly include antibodies and small-molecule tyrosine kinase inhibitors (TKIs). Bevacizumab (a macromolecular monoclonal antibody) can block tumor angiogenesis, which has been shown to be effective in metastatic breast cancer (11–14). Sorafenib, sunitinib and apatinib are anti-angiogenic TKIs that are mainly used to treat advanced liver cancer, metastatic renal cell carcinoma, metastatic gastric cancer, etc. (15–17). In terms of metastatic breast cancer, some clinical studies have also been carried out on anti-angiogenic TKI drugs. Sorafenib monotherapy could not prolong progression-free survival (PFS) in advanced breast cancer (18, 19). However, sorafenib combined with capecitabine could improve PFS in patients with HER2-negative advanced breast cancer (20). Sunitinib has a serious adverse event (AE) in the treatment of advanced breast cancer, so its application is limited (21). Apatinib monotherapy or combined with chemotherapy has shown efficacy in metastatic breast cancer (22–24).

Anlotinib is a novel oral anti-angiogenic TKI that blocking vascular endothelial growth factor receptor (VEGFR)1-3, fibroblast growth factor receptor (FGFR)1-4, platelet-derived growth factor (PDGFR)-α, PDGFR-β, and stem cell factor receptors, which inhibit tumor angiogenesis and tumor cell proliferation (25, 26). Many clinical studies have shown that anlotinib has encouraging efficacy and controllable toxicity in some solid tumors, such as non-small cell lung cancer, soft tissue sarcoma, small cell lung cancer, and medullary thyroid cancer (27–30). Preclinical studies have shown that anlotinib can inhibit the proliferation of breast cancer cells (31, 32). Anlotinib combined with TQB2450 (a humanized monoclonal antibody targeting programmed death-ligand 1) showed an acceptable safety profile and promising activity in advanced TNBC patients who were previously treated with anthracyclines and/or taxanes (33). A phase II clinical trial shows that anlotinib alone is effective for advanced breast cancer (34). A real-world study shows that single or combined treatment of anlotinib is effective for heavily pretreated HER2 negative metastatic breast cancer, with low toxicity (35).. These studies showed that anlotinib is effective in metastatic breast cancer, especially in HER2-negative subtypes or TNBC. However, to date, there is no prospective clinical study on the treatment of TNBC with anlotinib combined with chemotherapy. The purpose of this study was to evaluate the efficacy and safety of anlotinib in combination with chemotherapy of the physician’s choice in pretreated patients with metastatic TNBC. To our knowledge, this should be the first prospective report on the results of metastatic TNBC treated with anlotinib combined with chemotherapy.

## 2 Materials and methods

### 2.1 Patients

The current prospective study enrolled 40 Chinese female patients with pretreated metastatic TNBC who received anlotinib combined with chemotherapy at the National Cancer Center/National Clinical Research Center for Cancer/Cancer Hospital & Shenzhen Hospital, Chinese Academy of Medical Sciences and Peking Union Medical College during May 1, 2019 and April 30, 2022.

Eligible patients had to meet the following criteria (1): female patients ≥18 years old; (2) histologically confirmed TNBC (defined as ER negative and PR negative on immunohistochemistry [IHC] and negative HER2 status, defined as 0 or 1+ based on IHC; patients with HER2 2+ by IHC were subjected to a fluorescence *in situ* hybridization (FISH) test for the HER2 gene and the result was non-amplification) for the primary or metastatic lesion; (3) presence of at least one measurable metastatic lesion; (4) performance score of 0-1 according to the Eastern Cooperative Oncology Group (ECOG) scoring criteria; (5) relapsed or failed after previous anthracycline and/or taxane treatment in the (neo)adjuvant or metastatic setting; and (6) adequate organ function (mainly including liver function, kidney function, heart function, lung function, etc.). The exclusion criteria were as follows: (1) other malignant tumors have been diagnosed in the past 5 years; (2) abnormal laboratory test results or organ dysfunction; and (3) previously received treatment with anlotinib.

This study involving human participants was reviewed and approved by the institutional review boards and ethics committees (ethical code: 2019-33-2) of the National Cancer Center/National Clinical Research Center for Cancer/Cancer Hospital & Shenzhen Hospital, Chinese Academy of Medical Sciences and Peking Union Medical College and was conducted in accordance with the Declaration of Helsinki. All patients signed the written informed consent to participate in this study.

### 2.2 Study design and treatment

All patients participating in this study were treated with anlotinib (8 mg orally once daily) and a chemotherapeutic agent (oral vinorelbine, orally on days 1 and 8 of each 21-day cycle, with doses of 60 mg/m^2^ for the first cycle and 80 mg/m^2^ for the subsequent cycles; or albumin bound paclitaxel, 260 mg/m^2^ intravenously on day 1 of each 21-day cycle; or gemcitabine, 1000 mg/m^2^ intravenously on days 1 and 8 of each 21-day cycle; or eribulin, 1.4 mg/m^2^ intravenously on days 1 and 8 of each 21-day cycle; or capecitabine, 1000 mg/m^2^ orally twice daily on days 1 to 14 of each 21-day cycle; or oxaliplatin, 130 mg/m^2^ intravenously on day 1 of each 21-day cycle; or docetaxel, 75 mg/m^2^ intravenously on day 1 of each 21-day cycle).

The patients were followed up until October 31, 2022. At baseline and every two cycles (every 6 weeks) during treatment, tumor evaluation was conducted for evaluable lesions through computed tomography (CT) or magnetic resonance imaging (MRI).

The primary endpoint was progression-free survival (PFS), defined as the time from the start of oral anlotinib treatment to objective tumor progression or death from any cause, whichever occurred first. The secondary endpoints included OS (defined as the time from the start of treatment to the date of mortality from any cause), overall response rate (ORR, defined as the proportion of patients who achieved a confirmed complete response or confirmed partial response), clinical benefit rate (CBR, defined as the proportion of patients who achieved a confirmed complete response or confirmed partial response or stable disease for ≥ 24 weeks), disease control rate (DCR, defined as the proportion of patients who achieved a confirmed complete response or confirmed partial response or stable disease for ≥4 weeks), and safety. It should be noted that confirmed complete response/partial response were defined as complete response/partial response in at least 2 continuous tumor evaluation. The efficacy was evaluated in accordance with the Response Evaluation Criteria in Solid Tumors (RECIST) version 1.1, while the safety was assessed in accordance with the Common Terminology Criteria for Adverse Events (CTCAE), version 5.0.

### 2.3 Statistical analyses

All data were statistically analyzed using SPSS software (IBM Corp., Armonk, NY, USA; version 26.0) and GraphPad Prism 8 software (GraphPad Software, Inc., La Jolla, CA, USA). PFS and OS of patients were estimated by the Kaplan-Meier method. In addition, univariate and multivariate analyses were used to determine the impact of variables on PFS and OS by the Cox proportional hazards regression model. P-value < 0.05 was considered as statistically significant.

## 3 Results

### 3.1 Clinical characteristics

Forty patients with metastatic TNBC participated in this study. The baseline characteristics of patients are presented in **Table 1**. The median age at enrollment in the clinical study of the patients was 50 years (range from 26 to 72 years), and all patients were female. Sixteen patients (40.0%) had an ECOG performance status score of 0, and 24 patients (60.0%) had an ECOG performance status score of 1. Furthermore, 16 patients (40.0%) had grade I–II tumor histology, 24 (60.0%) had grade III tumor histology. A total of 23 patients (57.5%) had stage I–II disease at initial diagnosis, 17 patients (42.5%) had stage III-IV disease at initial diagnosis. Twenty-nine patients (72.5%) received one or two lines of treatment, and 11 patients (27.5%) received ≥ 3-line treatment. The majority of patients (30, 75.0%) had visceral metastasis, and 17 patients (42.5%) had more than 3 metastatic sites. All patients had received treatment with anthracycline and/or taxane before enrollment. In this study, the combined chemotherapeutic agents included oral vinorelbine (12, 30.0%), albumin bound paclitaxel (11, 27.5%), gemcitabine (9, 22.5%), eribulin (4, 10.0%), capecitabine (2, 5.0%), oxaliplatin (1, 2.5%) and docetaxel (1, 2.5%).

**Table 1:** Patient characteristics at baseline.

Characteristic	n (%)
Age of enrollment, years	
<50	20 (50.0)
≥50	20 (50.0)
Location	
Left	20 (50.0)
Right	20 (50.0)
ECOG performance status	
0	16 (40.0)
1	24 (60.0)
Histopathologic grade	
I-II	16 (40.0)
III	24 (60.0)
TNM stage at initial diagnosis	
I-II	23 (57.5)
III-IV	17 (42.5)
DFS duration, months	
≤24	27 (67.5)
>24	13 (32.5)
Lines of treatment, lines	
<3	29 (72.5)
≥3	11 (27.5)
Type of metastatic site	
Non-visceral	10 (25.0)
Visceral	30 (75.0)
Metastatic sites	
Liver	11 (27.5)
Lung	24 (60.0)
Bone	20 (50.0)
Brain	8 (20.0)
Number of metastatic sites, n	
≤3	23 (57.5)
>3	17 (42.5)
Previous chemotherapy	
Anthracycline	36 (90.0)
Taxane	39 (97.5)
Anthracycline or Taxane	40 (100.0)
Combined chemotherapeutic drug	
Oral vinorelbine	12 (30.0)
Albumin bound paclitaxel	11 (27.5)
Gemcitabine	9 (22.5)
Eribulin	4 (10.0)
Capecitabine	2 (5.0)
Oxaliplatin	1 (2.5)
Docetaxel	1 (2.5)

ECOG, Eastern Cooperative Oncology Group; TNM stage, the stage of tumor, node and metastasis; DFS, disease free survival.

### 3.2 Efficacy

**Figure 1:**
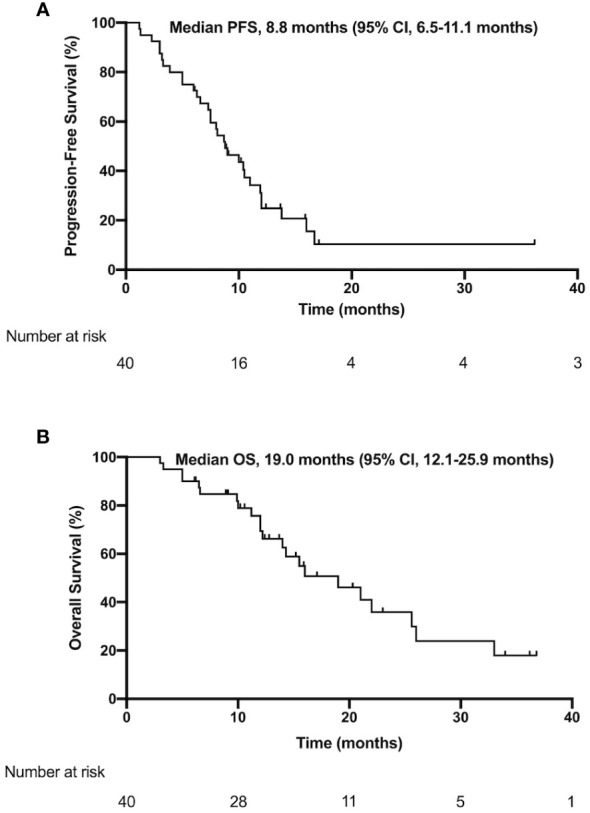
Kaplan–Meier estimates of PFS **(A)** and OS **(B)** in the patients with metastatic triple negative breast cancer who received anlotinib and chemotherapy. PFS, progression-free survival; OS, overall survival; CI, confidence interval.

The patients were followed up until October 31, 2022, and the median follow-up time was 12.6 months (range from 3.0 to 36.8 months). At the end of the follow-up, 31 patients discontinued the study treatment due to disease progression, no patients stopped the treatment permanently due to toxicity, 22 patients died from the disease progression, and no death caused by other reasons. As demonstrated in **Figure 1A** and **Table 2**, the median PFS was 8.8 months (95% CI, 6.5–11.1 months), and 9 patients still did not have disease progression at the last follow-up. The median OS was 19.0 months (95% CI, 12.1–25.9 months), and 18 patients were still alive to the end of follow-up (**Figure 1B**, **Table 2**).

**Table 2:** Efficacy outcomes (N=40).

Endpoint	
Primary endpoint	
Median progression-free survival, months (95% CI)	8.8 (6.5-11.1)
Secondary endpoints and other best clinical response	
Median overall survival, months (95% CI)	19.0 (12.1-25.9)
Complete response, no. (%)	0 (0)
Partial response, no. (%)	16 (40.0)
Stable disease, no. (%)	22 (55.0)
Disease progression, no. (%)	2 (5.0)
Objective response rate, no. (%)	16 (40.0)
Clinical benefit rate, no. (%)	34 (85.0)
Disease control rate, no. (%)	38 (95.0)

CI, confidence interval.

Among 40 patients, a total of 16 achieved PR as the best response, with an ORR of 40.0%. Thirty-eight patients achieved PR or SD, with a DCR of 95.0%. Additionally, 34 patients achieved PR or SD for more than 24 weeks, so the CBR of this study was 85.0%. None of the patients achieved CR (**Table 2**).

**Figure 2:**
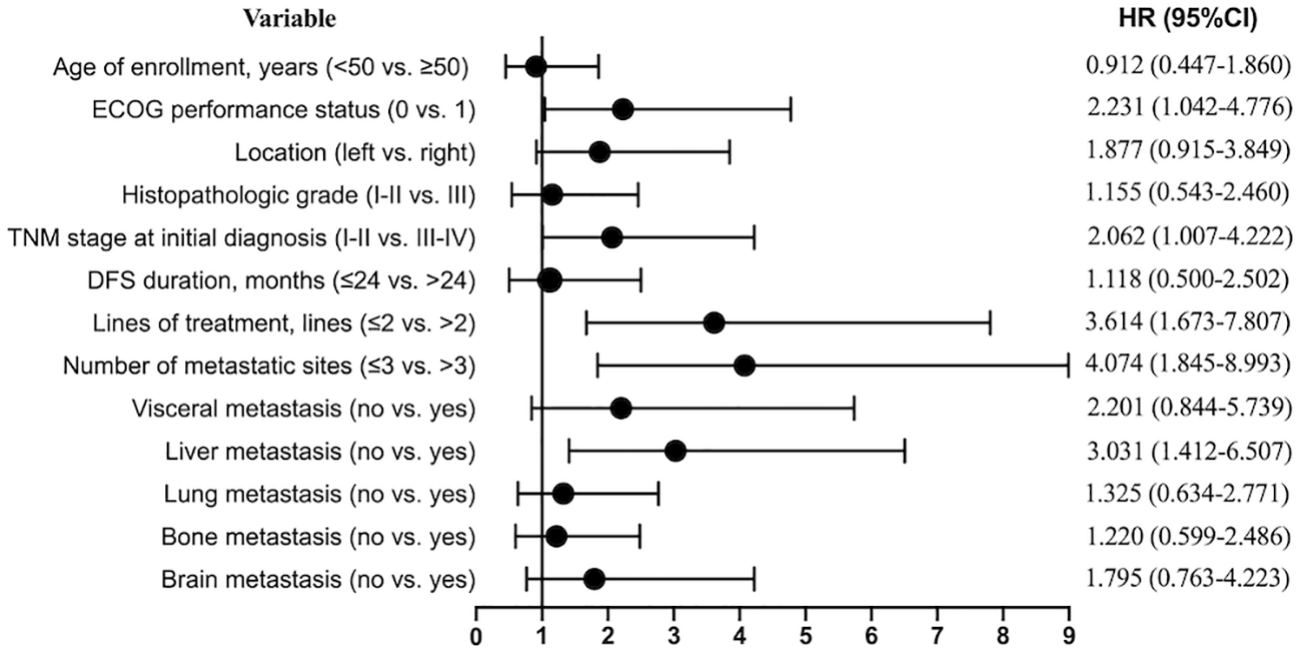
Forest plot of cox univariate regression analysis for progression-free survival. HR, hazard ratio; CI, confidence interval; ECOG, Eastern Cooperative Oncology Group; TNM stage, the stage of tumor, node and metastasis; DFS, disease free survival.

As presented in **Table 3** and **Figure 2**, univariate analysis of a total of 40 patients showed that ECOG performance status score of 1 (p = 0.039), stage III–IV disease at diagnosis (p = 0.048), received third-line or above treatment (p = 0.001), had more than 3 metastatic sites (p = 0.001), and had liver metastasis (p = 0.004) may exhibit a higher risk of disease progression.

**Table 3:** Cox univariate regression analysis for progression-free survival and overall survival.

	Progression-free survival			Overall survival		
Variable	HR	95% CI	P‐value	HR	95% CI	P‐value
Age of enrollment, years (<50 vs. ≥50)	0.912	0.447-1.860	0.800	1.111	0.475-2.598	0.809
ECOG performance status (0 vs. 1)	2.231	1.042-4.776	**0.039**	5.928	1.702-20.647	**0.005**
Location (left vs. right)	1.877	0.915-3.849	0.086	1.960	0.818-4.699	0.131
Histopathologic grade (I-II vs. III)	1.155	0.543-2.460	0.708	0.678	0.278-1.655	0.394
TNM stage at initial diagnosis (I-II vs. III-IV)	2.062	1.007-4.222	**0.048**	3.509	1.395-8.829	**0.008**
DFS duration, months (≤24 vs. >24)	1.118	0.500-2.502	0.786	1.331	0.498-3.556	0.569
Lines of treatment, lines (≤2 vs. >2)	3.614	1.673-7.807	**0.001**	4.802	1.784-12.925	**0.002**
Number of metastatic sites (≤3 vs. >3)	4.074	1.845-8.993	**0.001**	3.934	1.552-9.970	**0.004**
Visceral metastasis (no vs. yes)	2.201	0.844-5.739	0.107	1.217	0.406-3.642	0.726
Liver metastasis (no vs. yes)	3.031	1.412-6.507	**0.004**	4.314	1.731-10.756	**0.002**
Lung metastasis (no vs. yes)	1.325	0.634-2.771	0.455	0.703	0.293-1.691	0.432
Bone metastasis (no vs. yes)	1.220	0.599-2.486	0.584	1.714	0.723-4.061	0.221
Brain metastasis (no vs. yes)	1.795	0.763-4.223	0.180	2.548	1.006-6.453	**0.048**

HR, hazard ratio; CI, confidence interval; ECOG, Eastern Cooperative Oncology Group; TNM stage, the stage of tumor, node and metastasis; DFS, disease free survival.

Bold values indicate a p-value of < 0.05.

The univariate analysis (**Table 3**) indicated that the higher risk variables for death were as follows: ECOG performance status score of 1 (p = 0.005), stage III–IV disease at diagnosis (p = 0.008), third-line or above treatment (p = 0.002), more than 3 metastatic sites (p = 0.004), liver metastasis (p = 0.002), and brain metastasis (p = 0.048). The PFS, OS and corresponding 95% CIs for these factors that were statistical significant in univariate analysis are shown in **Table 4**.

**Table 4:** PFS and OS for subgroup analysis.

Characteristic	PFS (95% CI)	OS (95% CI)
ECOG performance status		
0	12.0 (11.0-13.0)	NE (NE-NE)
1	7.5 (3.8-11.2)	14.0 (9.1-18.9)
TNM stage at initial diagnosis		
I-II	11.0 (8.1-13.9)	33.0 (12.0-54.0)
III-IV	7.5 (3.7-11.3)	12.2 (9.6-14.8)
Lines of treatment, lines		
≤2	10.5 (9.0-12.0)	25.6 (18.2-33.0)
>2	5.0 (1.4-8.6)	12.0 (10.0-14.0)
Number of metastatic sites		
≤3	11.9 (9.9-13.9)	26.0 (12.4-40.0)
>3	6.3 (4.1-8.5)	12.0 (10.7-13.3)
Liver metastasis		
No	10.5 (9.0-12.0)	25.6 (18.7-32.5)
Yes	6.0 (2.7-9.3)	12.0 (10.1-13.9)
Brain metastasis		
No	10.0 (7.1-12.9)	21.0 (14.2-27.8)
Yes	6.3 (2.1-10.5)	12.0 (8.1-15.9)

PFS, progression-free survival; OS, overall survival; CI, confidence interval; ECOG, Eastern Cooperative Oncology Group; TNM stage, the stage of tumor, node and metastasis; NE, not evaulated.

**Figure 3:**
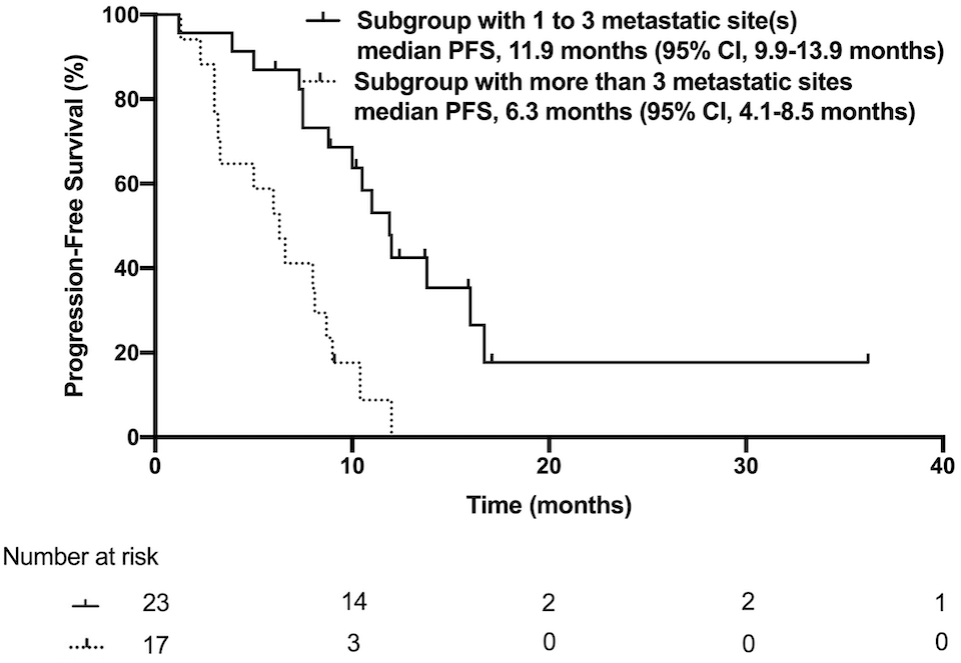
Kaplan–Meier estimates of progression-free survival in the subgroup with 1 to 3 metastatic site(s) or with more than 3 metastatic sites. PFS, progression-free survival; CI, confidence interval.

In addition, multivariate Cox analysis of variables with statistical significance in univariate analysis was conducted (**Table 5**). We carried out multivariate analysis on 5 factors influencing PFS in univariate analysis and found that having more than 3 metastatic sites (HR, 3.030; 95% CI, 1.193 to 7.692; p = 0.020) was an independent and meaningful unfavorable prognostic factor for PFS. The median PFS times were 6.3 months (95% CI, 4.1 to 8.5 months) in the subgroup with more than 3 metastatic sites and 11.9 months (95% CI, 9.9 to 13.9 months) in the subgroup with 1-3 metastatic site(s) (**Figure 3**). Multivariate analysis showed that there were no significant unfavorable prognostic factors for OS.

**Table 5:** Cox multivariate regression analysis for progression-free survival and overall survival.

	Progression-free survival			Overall survival		
Variable	HR	95% CI	P‐value	HR	95% CI	P‐value
ECOG performance status (0 vs. 1)	1.158	0.412-3.256	0.781	2.251	0.360-14.072	0.386
TNM stage at initial diagnosis (I-II vs. III-IV)	1.376	0.467-4.053	0.562	1.657	0.366-7.494	0.512
Lines of treatment, lines (≤2 vs. >2)	1.962	0.733-5.250	0.180	1.667	0.444-6.249	0.449
Number of metastatic sites (≤3 vs. >3)	3.030	1.193-7.692	**0.020**	1.993	0.615-6.464	0.250
Liver metastasis (no vs. yes)	1.094	0.349-3.426	0.877	1.414	0.411-4.864	0.583
Brain metastasis (no vs. yes)	–	–	–	1.395	0.440-4.427	0.572

HR, hazard ratio; CI, confidence interval; ECOG, Eastern Cooperative Oncology Group; TNM stage, the stage of tumor, node and metastasis.

Bold values indicate a p-value of < 0.05.

### 3.3 Safety

**Table 6** summarizes the treatment-related adverse events (TRAEs) that occurred in our study, including all grades. Among all 40 patients with toxicity records, 97.5% of patients (n=39) developed TRAEs of varying degrees and the incidence of grade 3-4 TRAEs was 37.5%. The non-hematological TRAEs included hand-foot syndrome (47.5%), secondary hypertension (45.0%), vomiting (40.0%), fatigue (40.0%), proteinuria (37.5%), diarrhea (37.5%), nausea (35.0%), oral mucositis (20.0%) and hemorrhage (5.0%). The hematological TRAEs were leukopenia (75.0%), neutropenia (70.0%), aspartate aminotransferase increase (20.0%), alanine aminotransferase increase (17.5%), thrombocytopenia (15.0%), hypertriglyceridemia (15.0%), anemia (12.5%) and hypercholesterolemia (12.5%). In addition, Grade 3-4 TRAEs were neutropenia (22.5%), leukopenia (20.0%), secondary hypertension (10.0%), hand-foot syndrome (5.0%), vomiting (5.0%), proteinuria (5.0%) and thrombocytopenia (2.5%). Most TRAEs were limited to patients with Grade 1-2 and were therefore tolerable and manageable. Two patients stopped taking anlotinib for 3 to 7 days due to grade 3 hand-foot syndrome and were able to continue taking anlotinib orally in subsequent cycles and tolerated the treatment well. None of the patients withdrew from the study because of treatment-related toxicity, and no deaths due to TRAEs occurred.

**Table 6:** Treatment-related adverse events.

Adverse events	All grade, n (%)	≥ Grade 3, n (%)
Non-hematologic		
Hand-foot syndrome	19 (47.5)	2 (5.0)
Secondary hypertension	18 (45.0)	4 (10.0)
Vomiting	16 (40.0)	2 (5.0)
Fatigue	16 (40.0)	0 (0)
Proteinuria	15 (37.5)	2 (5.0)
Diarrhea	15 (37.5)	0 (0)
Nausea	14 (35.0)	0 (0)
Oral mucositis	8 (20.0)	0 (0)
Hemorrhage	2 (5.0)	0 (0)
Hematologic		
Leukopenia	30 (75.0)	8 (20.0)
Neutropenia	28 (70.0)	9 (22.5)
Aspartate aminotransferase increase	8 (20.0)	0 (0)
Alanine aminotransferase increase	7 (17.5)	0 (0)
Thrombocytopenia	6 (15.0)	1 (2.5)
Hypertriglyceridemia	6 (15.0)	0 (0)
Anemia	5 (12.5)	0 (0)
Hypercholesterolemia	5 (12.5)	0 (0)

## 4 Discussion

As we know, this study should be the first prospective study to explore the activity and safety of anlotinib combined with chemotherapy in patients with metastatic TNBC. In this study, the median PFS of all 40 patients was 8.8 months (95% CI, 6.5–11.1 months), while the median OS was 19.0 months (95% CI, 11.8–26.2 months). In addition, the ORR was 40.0% (16/40), the DCR was 95.0% (38/40) and the CBR was 85% (34/40). These results indicated that the combination of anlotinib and chemotherapy has good activity in the treatment of metastatic TNBC.

Chemotherapy is very important for controlling the disease progression of patients with metastatic TNBC. The median PFS of first-line combined chemotherapy was between 5.5 months and 9.8 months (36–38). However, the efficacy is worse in patients with heavily pretreated metastatic TNBC, and a study showed that capecitabine combined with cisplatin in pretreated metastatic TNBC had a PFS of 3.68 months (39). 304 Study showed eribulin or vinorelbine were used as a multi-line treatment for patients with advanced breast cancer, the PFS was only 2.8 months (40). Therefore, the efficacy of chemotherapy alone (whether a combination of two drugs or a single-drug regimen) in the treatment of advanced TNBC is limited. In recent years, with the application of immunotherapy or PARP inhibitors combined with chemotherapy, the treatment efficacy of metastatic TNBC has been improved (41). Currently, patients with metastatic TNBC still have fewer treatment options than patients with other subtypes of breast cancer.

Anti-angiogenic drugs have shown certain efficacy in the treatment of some solid tumors. Studies have shown that the median PFS of bevacizumab, sorafenib, sunitinib and apatinib monotherapy for the treatment of advanced breast cancer was 2.0–4.0 months, and the ORR was 0%–16.7% (15, 19, 20, 22–24). In previous clinical studies, anlotinib monotherapy was also proven to be effective in the multi-line treatment of advanced breast cancer. Hu et al. (34) reported a phase II study of anlotinib monotherapy in pretreated HER2-negative metastatic breast cancer. Following the results, the median PFS of the population was 5.22 months, and the ORR was 15.38%. In subgroup analysis, the median PFS of TNBC patients was 4.04 months. It seems that the PFS of anlotinib monotherapy is longer than that of other anti-angiogenic drug monotherapies, but the efficacy of all anti-angiogenic drug monotherapies is still very limited. Anti-angiogenic drugs combined with chemotherapy may improve the effect of antitumor treatment in advanced breast cancer. The ORRs of bevacizumab, sorafenib, sunitinib and apatinib for the treatment of advanced breast cancer were significantly increased to 23.2%–51.3% after combination with chemotherapy, with median PFS of 4.4-11.8 months, and the result was better than monotherapy (12–14, 21, 25). In a real-world study of anlotinib monotherapy or combined with chemotherapy in multi-line therapy in patients with advanced breast cancer, the median PFS of monotherapy was 3.0 months, and that of combined treatment was 5.5 months. In subgroup analysis, the median PFS of TNBC patients was 3.5 months (35). In our study, the median PFS of TNBC patients who had previously received at least two lines of treatment in the metastatic setting was 5.0 months, which was longer than that of patients with metastatic TNBC reported by Hu et al. (4.04 months) (34) and Shao et al. (3.5 months) (35). In addition, the median PFS of anlotinib combined with chemotherapy as first-line or second-line treatment was 10.5 months, indicating that it was better than the existing reports on metastatic TNBC. Although these findings come from different study populations and evaluations, with consistent findings in metastatic TNBC, the combination of anlotinib and chemotherapy has good antitumor activity for early- or late-line treatment.

At the 2022 ASCO Annual Meeting, Liu et al. (42) reported a prospective clinical trial study of eribulin versus eribulin plus anlotinib in the treatment of patients with advanced or metastatic breast cancer. According to the published abstract results, the median PFS of patients with advanced TNBC treated with anlotinib plus eribulin reached 9.7 months. In addition, Yin et al. (43) also reported a single-arm phase II clinical study on the treatment of metastatic HER2 negative breast cancer with anlotinib and eribulin. However, the median PFS of this study was only 4.7 months. In our study, the median PFS of anlotinib combined with chemotherapy was 8.8 months, and the median PFS in the third-line treatment or above setting was 5.0 months. There were 11 patients with the third-line or beyond treatment (including 3 third-line patients, 1 fourth-line patient, 3 fifth-line patients, 3 sixth-line patients, and 1 tenth-line patient; all patients had visceral metastasis). Therefore, our study shows that anlotinib combined with chemotherapy has potential efficacy for TNBC patients were heavily pretreated and with visceral metastasis.

In our study, the most common TRAEs were leukopenia, neutropenia, hand-foot syndrome, secondary hypertension, vomiting, fatigue, proteinuria, etc. Among them, hematological toxicity and gastrointestinal reactions were mainly caused by chemotherapy drugs, while hand-foot syndrome, secondary hypertension and proteinuria were mainly caused by the anlotinib. The majority of TRAEs in patients receiving anlotinib combined with chemotherapy were grades 1-2, and the incidence was similar to that in previous relevant clinical trials (34, 35). Anti-angiogenic drugs are likely to increase the probability of hemorrhage. In our study, two patients suffered from hemorrhage, one from gum bleeding, and the other from chest wall tumor bleeding, both of which were very mild, without causing massive bleeding or anemia. No serious bleeding events were observed in the whole study, such as hemoptysis, gastrointestinal bleeding, hematuria and intracranial hemorrhage. RIBBON-2 trial showed that bevacizumab combined with chemotherapy could significantly prolong PFS of second-line treatment for patients with advanced breast cancer, but could not improve OS (13). In terms of safety, the AEs in the bevacizumab group that led to the discontinuation of the study were more than those in the placebo group (13.3% versus 7.2%), but in fact there is no difference in the number of treatment-related deaths between the two groups (6 patients in the bevacizumab group versus 5 patients in the placebo group) (13). In the subgroup analysis of TNBC, compared with placebo group, bevacizumab group could prolong PFS (6.0 months versus 2.7 months) and there is a trend to improve OS (17.9 months versus 12.6 months) (44). Two patients in both groups have treatment-related deaths (2% in bevacizumab group versus 4% in placebo group) (44). Similarly, in our study, anlotinib combined with chemotherapy showed potential efficacy and good tolerance. Only two patients (5%) temporarily stopped taking anlotinib due to grade 3 hand-foot syndrome (after active supportive treatment, their symptoms are relieved and they continued to take anlotinib orally), and there were no treatment-related deaths.

The current study is a small sample phase II clinical study, which from a single center in China. The limitation of this study is that it only enrolled a small number of patients and lacked a standard control group. However, anlotinib combined with chemotherapy is still a potential and effective alternative for patients with metastatic TNBC. We look forward to the conduction of more multicenter randomized controlled trials can be conducted in a larger cohort to further verify the efficacy and safety of anlotinib combined with chemotherapy.

## 5 Conclusions

In summary, the findings of this single-arm clinical trial showed that anlotinib combined with chemotherapy appeared to be efficacious for metastatic TNBC, with acceptable toxicity. It provides a potential and effective alternative for patients with metastatic TNBC.

## Data availability statement

The raw data supporting the conclusions of this article will be made available by the authors, without undue reservation.

## Ethics statement

The studies involving human participants were reviewed and approved by the institutional review boards and ethics committees (ethical code: 2019-33-2) of the National Cancer Center/National Clinical Research Center for Cancer/Cancer Hospital & Shenzhen Hospital, Chinese Academy of Medical Sciences and Peking Union Medical College and was conducted in accordance with the Declaration of Helsinki. The patients/participants provided their written informed consent to participate in this study.

## Author contributions

All authors contributed to the study conception and design. Material preparation, data collection and analysis were performed by J-YH, X-FX, X-LC, Q-YZ, L-PC and XB. Writing-reviewing and editing were performed by J-YH, X-FL, LS and J-FG. The first draft of the manuscript was written by J-YH and C-WD edited it critically. All authors reviewed the results and approved the final version of the manuscript. All authors contributed to the article and approved the submitted version.
